# Cabozantinib-Induced Severe Cardiac Dysfunction: A Case Report and a Systematic Review of the Literature

**DOI:** 10.7759/cureus.23740

**Published:** 2022-04-01

**Authors:** Chandan Buttar, Sofia Lakhdar, Mahmoud Nassar, Ian Landry, Most Munira

**Affiliations:** 1 Internal Medicine, Icahn School of Medicine at Mount Sinai, New York City Health and Hospitals/Queens, Jamaica, USA; 2 Internal Medicine, Queens Hospital Center, New York, USA; 3 Medicine, Icahn School of Medicine at Mount Sinai, New York City Health and Hospitals/Queens, Jamaica, USA; 4 Cardilogy/Medicine, Weil Cornell Medicine, New York, USA; 5 Cardiology, Queens Hospital Center, New York, USA

**Keywords:** heart failure, renal cell carcinoma, chemotherapy associated cardio toxicity, cardiomyopathy, cabozantinib

## Abstract

Cabozantinib is a novel multitargeted receptor tyrosine kinase inhibitor commonly used to treat advanced renal cell carcinoma. Cardiotoxicity is not a previously well-described adverse effect of cabozantinib. We present a rare case of a 74-year-old male with a history of renal cell carcinoma who underwent partial nephrectomy. The patient had been recently started on cabozantinib for advanced metastatic renal cell carcinoma. He developed acute onset of heart failure and subclinical hypothyroidism within nine months of treatment. Our case report postulates a causal relationship between cabozantinib and the development of non-ischemic cardiomyopathy.

## Introduction

Cardiotoxicity is the most severe side effect of cancer therapy, and it leads to increased patient morbidity and mortality [1]. The severity of toxicity depends on many factors, such as the molecular site of action, the immediate and cumulative dose, the method of administration, and the presence of underlying cardiac disease [1]. Many cancer survivors are as significantly at risk from cardiac disease as from recurrent cancer [2]. The treatment of metastatic renal cell carcinoma is rapidly evolving with the emergence of new targeted therapies and combination therapies. Advances in chemotherapeutic agents over the past few years have led to significantly improved cancer survival rates. Consequently, cardiac toxicity has emerged as a leading cause of morbidity during and after treatment. Early diagnosis is essential in this patient population, given the serious risk. We present a rare case of a patient with renal cell cancer who, based on our hypothesis, developed cardiac dysfunction secondary to the chemotherapeutic agent cabozantinib.

## Case presentation

This case involved a 74-year-old male with a history of hypertension, type 2 diabetes mellitus, chronic kidney disease stage III, non-obstructive coronary artery disease (CAD), and renal cell cancer status post right partial nephrectomy. Ten years later, he was found to have lesions in the liver concerning for metastasis. He was then started on cabozantinib 60 mg daily in June 2020. Transthoracic echocardiographic (TTE) evaluation at the time showed normal cardiac function, normal left ventricle (LV) wall contraction, normal valves, and left ventricular ejection fraction (LVEF) of 60%.

Eight months after initiating cabozantinib, the patient presented to the hospital following a sudden left-sided, non-radiating chest pain and dizziness. The patient was afebrile at 97.4 °F; he had a heart rate of 80 beats per minute (BPM), blood pressure of 149/80 mmHg, and was saturating at 100% on room air. He complained of dizziness that had been occurring for the past three weeks at rest and on exertion. Dizziness would last from a few minutes up to an hour. He also reported a reduction in exercise tolerance during this period. Physical examination was unremarkable and orthostatics were negative. An electrocardiogram (EKG) showed sinus rhythm with a heart rate of 80 with left ventricular hypertrophy (LVH). No findings from the physical examination were suggestive of acute heart failure at the time. Labs were significant for blood urea nitrogen (BUN) of 21 mg/dL and creatine of 1.98 mg/dL. The liver function test was within the normal range. Serial troponin-T tests were obtained and were negative. Pro B-type natriuretic peptide (pro-BNP) was 1,286 pg/mL. The patient was taking atorvastatin 40 mg nightly, carvedilol 25 mg twice daily, amlodipine 5 mg daily, glimepiride 1 mg daily, metformin 1000 mg twice daily, and cabozantinib 50 mg daily.

The echocardiogram on admission, as shown in Video 1, was concerning for severely decreased LVEF of 10-15% with global LV hypokinesia, biventricular dysfunction, and bilateral dilated ventricular cavities. Cardiac enzyme markers were negative. Repeat EKG was not significant for ischemia. Further workup for Coxsackie, Lyme disease, and HIV were negative. Both ferritin level and erythrocyte sedimentation rate (ESR) were within the normal range. Thyroid-stimulating hormone (TSH) was elevated to 17.20 compared to normal values eight months ago. LHC was negative for obstructive CAD.

**Video 1:** Limited echocardiogram demonstrating severely decreased ejection fraction with global left ventricular hypokinesia

The patient then underwent cardiac MRI (cMRI) one month later to rule out chemotherapy-induced cardiomyopathy. The cMRI showed mildly dilated LV with severely decreased systolic function and LVEF of 27%. No areas of late gadolinium enhancement suggested myocardial infarction, inflammation, or fibrosis. The patient was maintained on guideline-directed medical therapy for both CAD and heart failure. Repeat TTE three months later showed improvement in ejection fraction to 30-35%. The patient was then started on immunotherapy with nivolumab.

## Discussion

Systematic review

Methods

**Figure 1:**
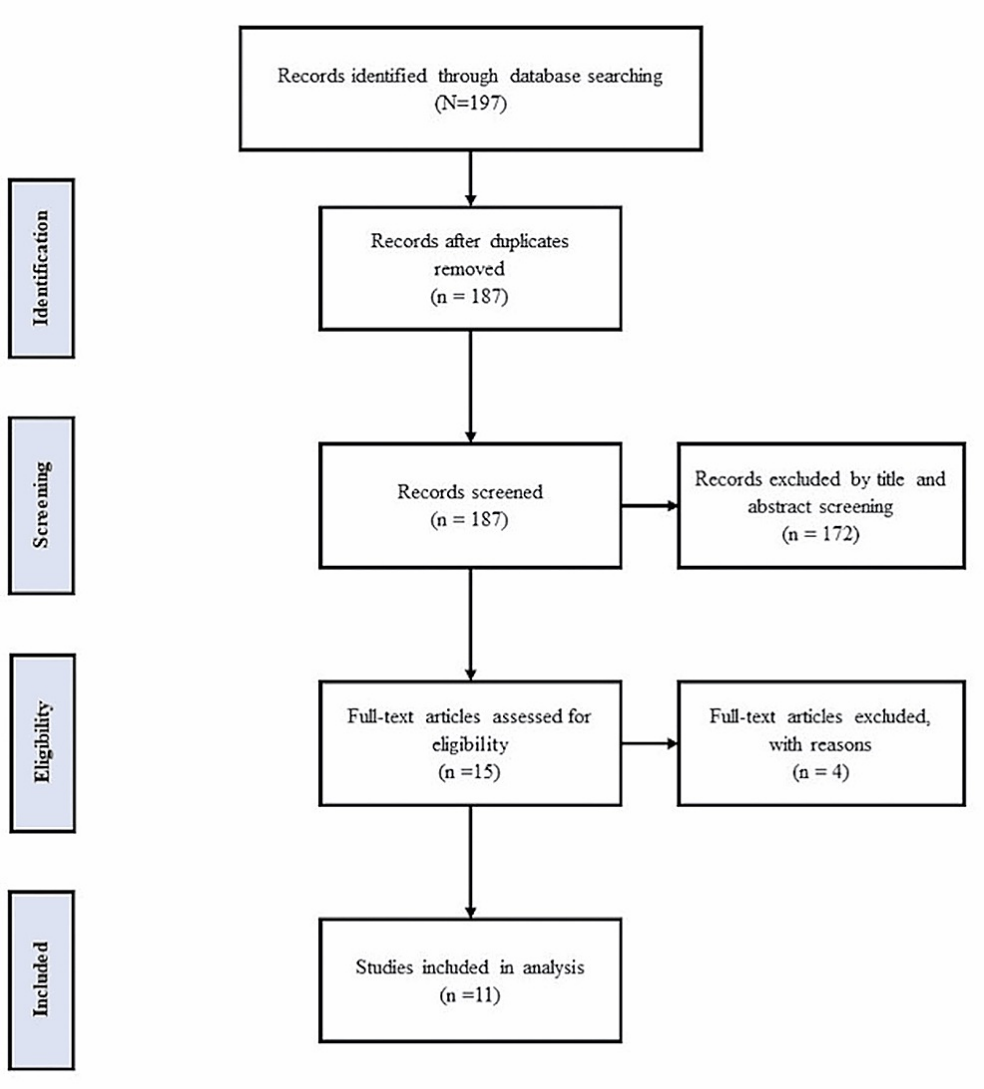
PRISMA flow diagram PRISMA: Preferred Reporting Items for Systematic reviews and Meta-Analyses

Our review involved a literature search that was conducted in December 2021 on the following databases: Embase, Medline, PubMed, and Web of Science. We used the keywords cabozantinib AND cardiovascular AND renal cell carcinoma. The inclusion criteria were case reports and case series. The exclusion criteria were non-peer-reviewed articles, pediatric- and pregnancy-related articles, and review articles. Articles were imported to the Covidence website and duplicated articles were removed. Two independent authors screened the articles. Data were extracted into a Microsoft Excel sheet. The descriptive analysis of the data was carried out using SPSS Statistics version 27 (IBM, Armonk, NY). Figure 1 shows the PRISMA flow diagram detailing the study selection.

Results

Data relating to 58 patients extracted from 13 studies were included in our review as seen in Figure 1. Seventeen patients had individual-level data, and data of 41 patients were abstracted from three separate studies that had aggregate-level data [3-5]. Table 1 shows the baseline patient demographics, tumor characteristics, and treatment-related adverse effects. Among the 17 individual patients, the mean age at diagnosis was 59 years old, whereas 41% of patients in the study by Prisciandaro et al. were less than 65 years old [3], and the median age at diagnosis in Iacovelli et al. was 67 years [4]. Nine (53%) of the individual 17 patients had at least one comorbid cardiac condition (i.e., hypertension, diabetes mellitus, dyslipidemia, prior CAD, or chronic kidney disease). The most common sites of metastatic lesions were lungs (59%), bone (47%), and lymph nodes (35%). All patients received cabozantinib 60 mg as first-line or subsequent therapy; 24 (41%) patients had baseline and subsequent TTE data available for review (Table 2).

**Table 1:** Patient demographics, tumor characteristics, and cardiac function of patients treated with cabozantinib Auvray et al. was an included study in an analysis by Perego et al. [12,13] PPE: palmar-plantar erythrodysesthesia; control: contralateral; ms: muscle; CAD: coronary artery disease; BPH: benign prostatic hyperplasia; SCC: squamous cell carcinoma; RP: retroperitoneal; PRES: posterior reversible encephalopathy syndrome; IVC: inferior vena cava; COPD: chronic obstructive pulmonary disease; CNS: central nervous system; CKD: chronic kidney disease; Inc: increased; LFT: liver function tests (aminotransferase); M: male; F: female

No.	Case	Patient/tumor characteristics				Treatment			
		Age, sex	Comorbidities	Metastases	Echo (LVEF %)	First-line	Subsequent lines	Dose reduction	Adverse effects
1	Prisciandaro et al. [3]	≤65 years old: 41%; >65 years old: 59%; male: 65%; female: 35%	Sites of metastases – lymph nodes: 53%; lungs: 59%; bone: 59%; liver: 24%	Previous anticancer therapy	2nd: cabozantinib (30%); 3rd: cabozantinib (11%); further: cabozantinib (59%)	40 mg (47%)	Any grade: 94%; grade 3/4: asthenia: 11%; diarrhea: 11%, Inc. LFTs: 6%; hypothyroidism: 6%; cardiac toxicity: 6%	Not reported	Not reported
2	Alhussein et al. [7]	70, M	Hypertension, dyslipidemia, atrial fibrillation, ischemic CAD	Stage IIIaN1	Base: 60; 2m: 25; 6m: 55	Pazopanib	Cabozantinib (60 mg)	20 mg	Heart failure
3	Tachibana et al. [14]	48, F	None	Lymph nodes, retroperitoneum	NA	Nivolumab, ipilimumab	Cabozantinib (60 mg)	20 mg	Grade 4: neutropenia; grade 2: hypothyroidism; grade 1: alopecia; grade 1: PPE; grade 1: diarrhea: grade 1: hoarseness
4	Tachibana et al. [14]	55, M	None	Lymph nodes, lungs, bone	NA	Nivolumab, ipilimumab	Cabozantinib (60 mg)	NA	Grade 2: diarrhea; grade 2: hypothyroidism; grade 2: hypertension; grade 1: PPE
5	Tachibana et al. [14]	36, M	History of seminoma	Lymph node, Control kidney, iliopsoas ms	NA	Nivolumab, ipilimumab	Cabozantinib (60 mg)	NA	Grade 2: diarrhea; grade 2: hypothyroidism; grade 2: hypertension; grade 1: PPE
6	Zarling et al. [15]	76, M	Hypertension, diverticulosis, BPH, SCC of the ear	RP lymph nodes	NA	Pazopanib	2nd: nivolumab; 3rd: everolimus; 4th: cabozantinib; 5th: nivolumab with ipilimumab	20 mg	Hypertension; hand-foot syndrome
7	de Velasco et al. [16]	60, M	None	IVC, renal vein, paraaortic lymph node, lung	NA	Cabozantinib	NA	NA	None
8	de Velasco et al. [16]	46, F	None	Lymph nodes, r-clavicle, lung	NA	Cabozantinib	NA	40 mg	Grade 2: mucositis
9	de Velasco et al. [16]	59, M	Current smoker, COPD	Lung, r-iliac crest	NA	Cabozantinib	NA	NA	None
10	de Velasco et al. [16]	60, M	Hypertension, diabetes mellitus, CAD	Lung	NA	Cabozantinib	NA	40 mg	Grade 2: asthenia, dysphonia, diarrhea, anxiety
11	Patwari et al. [17]	70, F	CKD, hypertension	Thoracic spine	NA	Nivolumab, ipilimumab	Nivolumab alone; cabozantinib	Discontinued (adverse effects)	PRES syndrome
12	Tucker et al. [18]	64, F	None	IVC, thoracic spine, lung	NA	Cabozantinib	Nivolumab	Discontinued (progression)	None
13	Bilen et al. [19]	59, M	None	Thoracic spine, psoas ms, tail of pancreas, splenic flexure, inferior spleen, abdominal wall	NA	Cabozantinib	NA	40 mg; 20 mg	Hypertension; hand-foot syndrome
14	Kao et al. [20]	48, M	Diabetes mellitus, hypertension, dyslipidemia	Rib cage	NA	Interleukin 2	2nd: sunitinib/pazopanib; 3rd: cabozantinib; 4th: nivolumab with 20 mg cabozantinib	40 mg; 20 mg	Grade 2: mucositis, fatigue, chelitis
15	Stellato et al. [21]	66, M	None	Bone, lung, heart	NA	Sunitinib	Cabozantinib	NA	None
16	Negrier et al. [22]	51, M	Hypertension	Lung	NA	Sunitinib	2nd: everolimus; 3rd: axitinib; 4th: nivolumab; 5th: cabozitinib	40 mg	Fatigue, stomatitis, weight loss
17	Négrier et al. [22]	55, M	Hypertension	CNS, lung, scalp	NA	Pazopanib	Cabozantinib	40 mg	Grade 3: diarrhea
18	Lakhdar et al. (our patient)	74, M	Hypertension, diabetes mellitus, CKD, non-obstructive CAD	***	Base: 60; 8m: 35/15; 10m: 25	Cabozantinib	Nivolumab	60 mg	***

**Table 2:** Assessment of cardiac function in patients treated with cabozantinib ^1^The patient received two echocardiograms during an admission at 8 months of starting cabozantinib. ^2^Echocardiogram was done at 10 months of starting cabozantinib LVEF: left ventricular ejection fraction; mo: months; CAD: coronary artery disease; CABG: coronary artery bypass graft

Author	Case no.	Echocardiogram (LVEF %)			Cardiovascular comorbid conditions (before cabozantinib)
		Baseline	At 3 months	At 6 months	
Iacovelli et al. [4]	1	64	65	61	Hypertension: 72.7%; CAD (ischemic): 9.1%; arrhythmia: 9.1%
	2	64	N/A	N/A	
	3	60	60	67	
	4	69	66	66	
	5	70	60	47	
	6	57	56	N/A	
	7	52	55	61	
	8	66	60	N/A	
	9	62	62	67	
	10	51	51	66	
	11	67	N/A	47	
	12	60	N/A	N/A	
	13	70	N/A	N/A	
	14	61	N/A	58	
	15	66	N/A	64	
	16	60	N/A	64	
	17	60	N/A	N/A	
	18	60	N/A	N/A	
	19	55	N/A	N/A	
	20	55	N/A	N/A	
	21	60	N/A	N/A	
	22	60	N/A	N/A	
Alhussein et al. [7]		60	25	55	Hypertension, dyslipidemia, atrial fibrillation, CAD, history of CABG
Lakhdar et al. (our patient)		60	35, 15^1^	25^2^	Hypertension, diabetes mellitus, chronic kidney disease, CAD non-ischemic

Analysis

Cabozantinib is a multitargeted receptor tyrosine kinase inhibitor with potent activity against MET and vascular endothelial growth factor 2 (VEGFR2). It has been known to prolong overall survival. Cabozantinib-induced cardiomyopathy has not been commonly seen and only two cases have been reported so far [6-7]. A literature search showing a reversible reduction in LVEF from cabozantinib was demonstrated in one of the case reports. Our patient had normal LV function prior to cabozantinib exposure. The most frequent cardiotoxicity side effects of chemotherapy drugs are myocardial ischemia and infarction. Cardiomyopathy, myopericarditis, and arrhythmias are commonly seen with the use of anthracyclines [8]. Heart failure, myopericarditis, and arrhythmias are also seen in patients treated with cyclophosphamide, and taxanes were found to be associated with heart failure, ischemia, and arrhythmias [8]. Cabozantinib is highly protein-bound in human plasma (>99.7%) and is a substrate of cytochrome P450 3A (CYP3A) [9]. In this case, the patient's medications were also reviewed to identify any CYP-mediated drug interactions; however, the patient was not on any medication that could affect the efficacy or toxicity of cabozantinib. Nonetheless, this patient had known chronic kidney disease; some studies have suggested that cabozantinib should be used with caution in subjects with mild or moderate renal impairment. Both hepatic and renal disease are intrinsic factors that may affect the absorption, metabolism, protein binding, and elimination of orally administered anticancer drugs [9]. In this patient, with a GFR of 32 ml/min, BUN of 21 mg/dL, and creatine of 1.98 mg/dL, it is not impossible that the impaired renal function increased the risk of toxicity.

Our patient was also found to have an elevated TSH, which initially had been normal prior to starting cabozantinib. Patients with a TSH level >10 mIU/L have been found to have a higher risk of developing heart failure with reduced ejection fraction as compared to subjects with normal thyroid function [10,11]. In addition, hypothyroidism can result in decreased cardiac output, increased systemic vascular resistance, decreased arterial compliance, and atherosclerosis [11-13]. Heart failure in this patient may have been a direct effect of cabozantinib or indirectly related due to the development of cabozantinib-induced thyroid dysfunction.

Assessment of Cardiac Function

Iacovelli et al. performed a multicenter study evaluating the cardiac function of 22 patients treated with cabozantinib [4]. In this study, reduced ejection fraction was defined as LVEF <55%. At baseline, 9% of patients had reduced LVEF, but none was with diastolic dysfunction; 18 of the 22 patients were assessed for follow-up at three months with cabozantinib, with 33.3% of patients experiencing a decline in their systolic function, and 11.1% experiencing a decline of more than 10%. Seven patients were assessed at six months of follow-up, and only one patient had a >10% decline in the baseline function. At baseline, pro-BNP and high-sensitivity troponin (hsTnl) were elevated in 64% and 27% of patients, respectively. However, no correlation was found between hsTnl (R=0.45, p=0.59) or elevation of pro-BNP (R=0, p=1.0) and reduction in LVEF.

Alhussein et al. have reported the case of a patient who underwent a dose reduction due to heart failure [7]. At baseline, the patient had a normal LVEF of 60% and then developed signs of heart failure after two months of treatment with cabozantinib. Repeat TTE showed an ejection fraction of 20-25%. Cabozantinib was stopped, and follow-up TTE at six weeks and 14 weeks showed improvement in LVEF to 34% and 50-55%, respectively. Our patient also had significantly reduced ejection fraction (60% to 30-35%, and to 10-15%) following eight months of treatment with cabozantinib. After the cessation of treatment, repeat TTE showed mild improvement in LVEF to 20-25%.

Adverse Effects

Our analysis found that 12 out of 17 patients (70%) had their dose of cabozantinib reduced or discontinued due to adverse effects or progression of the disease. The most common adverse effects were diarrhea (29%), hypertension (23.5%), asthenia/weakness (17.6%), and hypothyroidism (17.6%). In comparison, Prisciandaro et al. found that 11% of patients had diarrhea, 11% had asthenia/weakness, and 6% had hypothyroidism [3]; 47% of patients in their study had their dose reduced, and 94% of patients experienced an adverse effect of some grade [3].

## Conclusions

Based on our findings, cabozantinib is a promising agent for the treatment of renal cell carcinoma as well as other cancers. It may, however, carry a significant risk of cardiotoxicity, which requires monitoring before, during, and after the treatment. Therefore, it should be used cautiously, especially in those with cardiac risk factors. Nonetheless, as previously mentioned, further studies are required to gain more insights into the cardiovascular safety profile of cabozantinib.
